# Only a minority of broad-range detoxification genes respond to a variety of phytotoxins in generalist *Bemisia tabaci* species

**DOI:** 10.1038/srep17975

**Published:** 2015-12-10

**Authors:** Eyal Halon, Galit Eakteiman, Pnina Moshitzky, Moshe Elbaz, Michal Alon, Nena Pavlidi, John Vontas, Shai Morin

**Affiliations:** 1 Department of Entomology, the Hebrew University of Jerusalem, Rehovot 76100, Israel; 2 Department of Biology, University of Crete, Heraklion, Crete 71409, Greece; 3 Institute of Molecular Biology & Biotechnology, Foundation for Research & Technology Hellas, Heraklion, Crete, Greece; 4 Laboratory of Pesticide Science, Department of Crop Science, Agricultural University of Athens, Athens, Greece

## Abstract

Generalist insect can utilize two different modes for regulating their detoxification genes, the constitutive mode and the induced mode. Here, we used the *Bemisia tabaci* sibling species MEAM1 and MED, as a model system for studying constitutive and induced detoxification resistance and their associated tradeoffs. *B. tabaci* adults were allowed to feed through membranes for 24 h on diet containing only sucrose or sucrose with various phytotoxins. Quantitative real-time PCR analyses of 18 detoxification genes, indicated that relatively few transcripts were changed in both the MEAM1 and MED species, in response to the addition of phytotoxins to the diet. Induced transcription of detoxification genes only in the MED species, in response to the presence of indole-3-carbinol in the insect’s diet, was correlated with maintenance of reproductive performance in comparison to significant reduction in performance of the MEAM1 species. Three genes, *COE2*, *CYP6-like 5* and *BtGST2,* responded to more than one compound and were highly transcribed in the insect gut. Furthermore, functional assays showed that the *BtGST2* gene encodes a protein capable of interacting with both flavonoids and glucosinolates. In conclusion, several detoxification genes were identified that could potentially be involved in the adaptation of *B. tabaci* to its host plants.

In the co-evolution theory proposed by Ehrlich and Raven in 1964^1^, the intimate interaction between plants and their insect herbivores is described as a dynamic system, subject to continual variation and change. In order to reduce insect attack, plants develop a wide-range of defense mechanisms which considerably alter their chemical and physical characteristics^2^,^3^. In parallel, insects develop multiple behavioral and chemical mechanisms to overcome plant defenses^4^. Among these, the ability to metabolize (detoxify) phytotoxins (allelochemiclas) is considered one of the major mechanisms insect have evolved for handling the vast diversity of plant toxins present in their diet^5^.

Insect phase I and phase II detoxification systems typically include five main enzyme families: cytochrome P450 monooxygenases (P450s or *CYPs* for genes), glutathione S-transferases (GSTs), carboxylesterases (COEs), UDP-glucosyltransferases (UGTs) and sulfotransferases^4^,^6^. Phase I enzymes, like P450s and COEs, introduce reactive and polar groups into their substrates through oxidation, hydrolysis or reduction^7^,^8^. Following phase I, the activated metabolites are conjugated with compounds such as glutathione, sulphate or glycosyl group in phase II reactions, to increase their hydrophilicity and excretion efficiency^9^.

Polyphagous (generalists) and oligophagous (specialists) insect herbivores interact with their host plants in very different ways. Whereas generalists are able to develop on a broad range of host plants, typically spanning several plant families, specialists have much more restricted host ranges^10^,^11^. Moreover, generalist herbivores typically possess enzyme systems capable of detoxifying a broad range of plant defensive chemicals (general detoxifiers), including novel chemistry they have never encountered^12^,^13^, albeit not as efficiently as specialist herbivores^14^. Specialist herbivores, on the other hand, typically possess enzyme systems that are highly specific and efficient at detoxifying specific plant defensive compounds characteristic of their narrow range of host plants^15^.

A fundamental issue about which there seems to be much uncertainty concerns the transcriptional regulation of detoxification genes. In general, insect herbivores may utilize two different modes (strategies) for regulating genes coding for enzymes involved in detoxification resistance to plant defensive compounds: the constitutive mode, in which the detoxification genes are transcribed independent of encountering a defended plant, and the induced mode, in which the detoxification genes are activated only after contact with the plant toxic chemistry^16^. Despite the fact that transcriptional regulation of detoxification enzymes in response to dietary constituents should be particularly challenging for broadly polyphagous species, which can encounter dozens of biosynthetically distinct toxins across their host range^13^, induced detoxification resistance against phytotoxins was speculated to be the more widespread phenomenon in generalist herbivores, whereas constitutive resistance was considered to be rare and restricted to specialists^4^.

The whitefly, *Bemisia tabaci* (Gennadius) (Hemiptera: Aleyrodidae) is a phloem-feeding insect that lives predominantly on herbaceous species. It is considered an extreme generalist and a major pest of ornamental, vegetable, grain legume, and cotton production, causing damage directly through feeding and indirectly through the transmission of plant pathogenic viruses^17^,^18^. *B. tabaci* has been recognized as a complex of 11 well-defined high-level generic groups containing as many as 34 morphologically indistinguishable species^19^,^20^ that differ in their biological characteristics^21^. The two most widespread species are Middle East Asia Minor 1(MEAM1 – previously called B) and Mediterranean (MED – previously called Q), which belong to separate but closely related clades^19^.

Several characteristics make the *B. tabaci* complex of species (and particularly MEAM1 and MED) a promising model system for studying the relationship between constitutive and induced detoxification resistance to plant phytotoxins in generalist insects. First, both MEAM1 and MED are extremely polyphagous and have a broad host plant range which includes edible and ornamental crops in both field and greenhouses^22^,^23^. Second, most research on detoxification resistance in generalist has focused so far on chewing insects. Phloem-feeders, on the other hand, cause a less drastic, more subtle defense response in plants and only experience defensive secondary metabolites that are translocated in the phloem^24^. Moreover, they often suppress more plant defense genes than the chewing herbivores, suggesting that they can minimize the activation of plant defenses^25^. In addition, the advantages of induced detoxification in phloem feeders might be different from that of chewing insects for the fact that defensive plant compounds occur in the phloem at a level that cannot necessarily be compared to that which a leaf chewer might be exposed to. Hard data to support this contention is scarce as minimal versus maximal exposures in the comparison between phloem feeders and leaf chewers remains speculative. Therefore, the importance/advantage of induced detoxification in successful host utilization seems not to be clear at the moment for this feeding guild. Third, several lines of evidence suggest that the MEAM1 and MED species of *B. tabaci* went through an allopatric divergence process^21^, in which they were likely to encounter different plant hosts and therefore, host-related selection forces leading to differences in tolerance to plant defenses^19^. Fourth, in a previous study^26^, we identified significant differences in the transcription profiles of several orthologous detoxification genes in MEAM1 and MED individuals when subjected to *Brassica* plant hosts.

Our overall goal here was to study various aspects of the relationship between constitutive and induced detoxification resistance to phytotoxins in *B. tabaci*. Our analyses concentrated on a set of eighteen detoxification genes, believed to play a key role in *B. tabaci* detoxification responses to environmental stress, due to their recurrent appearance in our previously reported cDNA libraries of strains showing susceptibility or resistance to several groups of chemical insecticides and to flavonoids^27^,^28^. Moreover, centering on this carefully selected set of genes allowed us not only to correlate specific gene transcription with the presence of a specific phytotoxin in the insect diet, but also to get a broader and reliable perspective on their general transcriptional “behavior” under multiple conditions. We focused on four main topics: (I) The extent to which detoxification genes of *B. tabaci* (MEAM1 and MED) are induced in response to the presence of various phytotoxins in the insect diet? (II) How specific is the induced response? In other words, which mechanism rules: “specific gene/s for specific phytotoxin” or “few detoxifiers for many phytotoxins”? (III) How much do MEAM1 and MED differ in their constitutive and induced transcription of detoxification genes? Can these differences be related to ecological traits such as reproductive performance? (IV) Whether the induced transcription of detoxification genes (in response to the presence of phytotoxins in the insect diet) might be related to a possible detoxification role of the encoded enzymes?

## Results

### Constitutive and induced transcription

We compared the transcription levels of eighteen detoxification genes in MEAM1 and MED adults, after feeding for 24 h on sucrose diets containing or lacking six plant phytotoxins: nicotine and caffeine (nicotine and purine alkaloids, respectively), flavone (flavone aglycone) and quercetin (flavonol aglycone), indole-3-carbinol (I3C) and allyl-isothiocyanate (AITC) (indolyl and aliphatic isothiocyanates, respectively).

**Figure 1:**
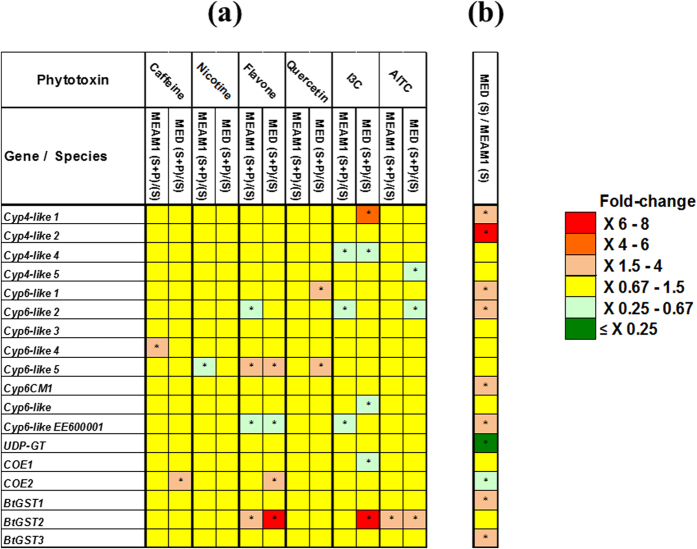
Transcription profiles of 18 detoxification genes from the P450, UGT, COE and GST families, after exposure of the MED and MEAM1 species to caffeine, nicotine, flavone, quercetin, I3C and AITC phytotoxins in sucrose diet. (**a**) Adults were fed for 24 h on sucrose diet containing the different phytotoxins. Transcription levels are shown as mean fold-transcription levels when feeding on sucrose diet containing the phytotoxin (S + P) relative to ‘sucrose only’ (S) in each *B. tabaci* species separately. (**b**) Relative expression of the 18 detoxification genes in the two species (MED/MEAM1), after feeding for 24 h on ‘sucrose only’ diet. Red and green spectrum indicate significant over- and under-transcription, respectively (ratio > 1.5-fold in either direction). Asterisks indicate significant differences (one-way ANOVA model, *P* ≤ 0.05). Yellow indicates no significant transcription differences.

The outcome of our analyses can be grouped into six major findings (comprehensive data of all statistical analyses are summarized in Table S1 and Table S2). First, in general, relatively few genes were induced in both species in response to the addition of phytotoxins to the sucrose diet (the “heat map” in Fig. 1a is dominated by yellow squares indicating no transcription differences within species). Second, when the constitutive transcription levels of the detoxification genes were compared between MEAM1 and MED adults feeding on sucrose diet lacking phytotoxins (‘sucrose only’), it was found that eight of the 18 genes were transcribed significantly higher in MED while only two genes were transcribed significantly higher in MEAM1 (Fig. 1b). Third, we were able to identify genes that were specifically up-regulated only in one of the species, indicating that the MEAM1 and MED species may differ in promoter regulatory elements responsible for induced responses. For example, the induction of *CYP4-like 1* by I3C and *CYP6-like 1* by quercetin in MED and the induction of *CYP6-like 4* by caffeine in MEAM1 (Fig. 1a). Fourth, at least three genes were shown to respond to more than one compound. This includes the induction of *BtGST2* by I3C in the MED species, and by flavone and AITC in both species, the induction of *COE2* by caffeine and flavone in the MED species and the induction of *CYP6-like 5* by flavone in both species and by quercetin in the MED species (Fig. 1a). Fifth, we were able to identify genes, like *CYP4-like 1* and *CYP6-like 1* (which are induced only in MED by I3C and quercetin, respectively), which also differ in their constitutive transcription levels between the two species (Fig. 1a,b). This finding may indicate that the regulatory regions of these genes differ both in their constitutive and induced elements between the two species. Sixth, the transcription of several *CYP* genes and *COE1* was down-regulated after 24 h of exposure to some phytotoxins (green squares in Fig. 1a).

### Induction of detoxification genes in MED is associated with the maintenance of reproductive performance

**Figure 2:**
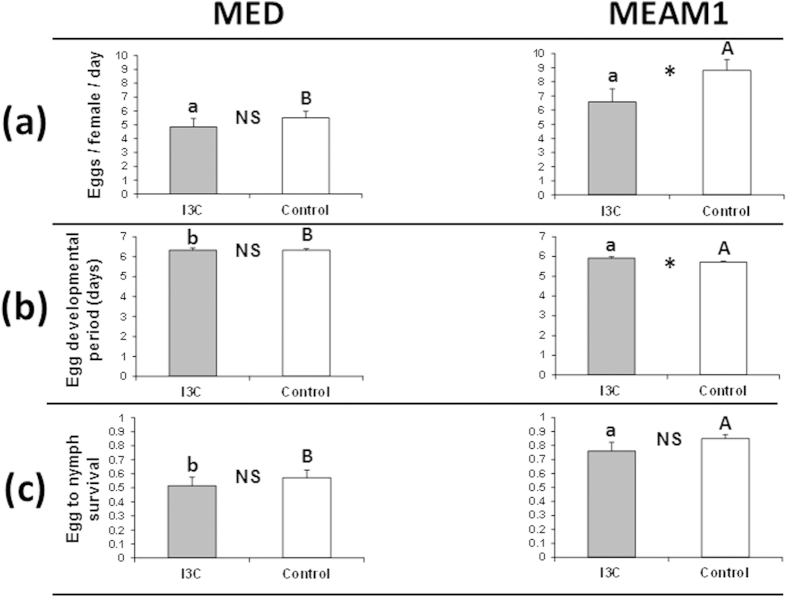
Reproductive performance of the MED and MEAM1 species of *B. tabaci* after feeding for 24 h on sucrose containing I3C or ‘sucrose only’ diets. (**a**) Oviposition rate (eggs/female/day). (**b**) Egg developmental period (days). (**c**) Proportion of eggs that hatched after eight days. Asterisks and different letters indicate significant differences (*P* ≤ 0.05). Errors bars represent standard error of the means (*n* = 15 for each species X treatment combination). N.S. = Not significant. Lower case letters = Interspecific comparisons on sucrose diet containing I3C. Upper case letters = Interspecific comparisons on ‘sucrose only’ diet. Asterisks = Intraspecific comparisons.

The highest induction of detoxification genes was observed in MED when subjected to a sucrose diet containing I3C. Two genes, *CYP4-like 1* and *BtGST2,* were specifically up-regulated (5.66-fold, *P* = 0.0018 and 6.13-fold, *P* = 0.0038, respectively) after 24 h of feeding on I3C, while their transcription levels remained un-changed in MEAM1 (Fig. 1a). Moreover, eight of the 18 analyzed genes showed constitutive higher transcription in MED than in MEAM1 (Fig. 1b). To test whether the observed differential gene transcription can be related to ecological traits such as reproductive performance, the number of oviposited eggs, their developmental period and survival were recorded for four days in MED and MEAM1 females after completing a 24 h feeding period on ‘sucrose only’ or sucrose plus I3C diets. Estimations of two independent life-history traits related to reproduction, indicated reduced performance of MEAM1 after feeding on sucrose diet containing I3C relative to ‘sucrose only’ diet. The number of oviposited eggs per day per female was significantly lower (*P* = 0.031) and the egg to first nymph developmental period was significantly longer (*P* = 0.015) in females that fed on sucrose plus I3C diet (Fig. 2a,b). Although egg to first nymph survival was also lower in MEAM1 females that fed on sucrose diet containing I3C relative to ‘sucrose only’ diet, this difference was not significant (*P* = 0.28) (Fig. 2c). In contrast, none of the three reproductive traits differed between MED females that were fed on ‘sucrose only’ or sucrose plus I3C diets (*P* ≥ 0.48) (Fig. 2a–c). Interspecies comparisons indicated that the number of oviposited eggs per day per female was significantly higher in MEAM1 (relatively to MED) after feeding on ‘sucrose only’ diet (*P* = 0.0009) but not after feeding on sucrose diet containing I3C (*P* = 0.124) (Fig. 2a). On the other hand, the egg to first nymph developmental period was significantly shorter and the egg to first nymph survival was significantly higher in progeny of MEAM1 females (relative to MED) that fed on sucrose diet containing I3C or ‘sucrose only’ diets (*P* ≤ 0.003) (Fig. 2b,c). To our best understanding, these performance data hold two complementary findings. Overall, and regardless of the diet used, the reproductive performance of the MEAM1 species outcompetes that of the MED species. At the same time, only the MEAM1 species shows relative reduced performance when facing a diet containing toxic indolyl-glucosinolates while the MED species performance remains unaffected (see discussion part below).

### Genes responding to more than one compound are highly transcribed in the insect’s gut

**Figure 3:**
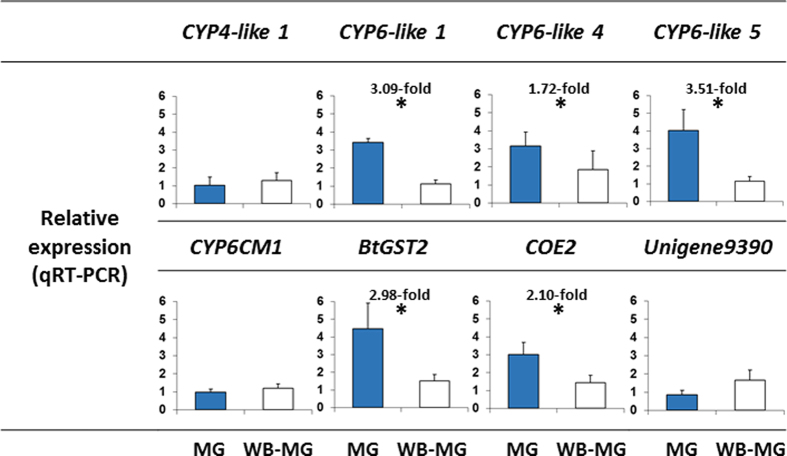
Expression of gut specific genes. Total RNA was separately extracted from the gut (MG) and the rest of the body (WB-MG) of MED females. The relative expression of seven detoxification genes and *Unigene9390* (a gene reported to be transcribed in the salivary gland) was tested by qRT-PCR. Genes were considered significantly over- or under-transcribed when the ∆CT values of samples from the gut were different from ∆CT values of samples from the rest of the body at *P* ≤ 0.05.Values presented are mean 2^−ΔΔct^ ± SE. Asterisks indicate significant differences (one-way ANOVA model).

Another important question relates to transcription patterns (localization) of detoxification genes within the insect body. As a major organ of the insect digestive system, the gut is likely to serve as a first barrier, involved in the detoxification of phytotoxins and xenobiotics harmful substances present in the insect diet^29^. Here, we tested the possibility that detoxification genes that are induced by the presence of phytotoxins in the insect diet (and among them, the three genes identified in the previous section, *COE2*, *CYP6-like 5* and *BtGST2,* which responded to more than one compound), are highly transcribed in the gut tissue. Total RNA was separately extracted from the gut and the rest of the body of MED females. qRT-PCR results showed that all induced genes (with the exception of *CYP4-like 1*) were significantly over-transcribed in the gut relative to the rest of the insect body (0.0018 ≤ *P* ≤ 0.045) (Fig. 3). In contrast, the transcription of the two selected control genes, *CYP6CM1* (a non-induced P450 gene shown to be involved in neonicotinods detoxification) and *Unigene9390* (a gene reported to be transcribed in the salivary gland^30^) was not different between the gut and the rest of the body (*CYP6CM1*, *P* = 0.82; *Unigene9390*, *P* = 0.28) (Fig. 3).

### Identifying inhibitors and substrates of BtGST2

*BtGST2* was the most versatile and inducible gene in our analyses. The gene was induced by I3C in the MED species, and by flavone and AITC in both species (Fig. 1a). In order to accumulate initial evidence for the putative involvement of the BtGST2 enzyme in detoxification (GSH conjugation) of plant phytotoxins, the coding sequence was cloned in the pET 100/D-TOPO expression vector (Invitrogen Life Technologies) and expressed in *E. coli*. The successful purification of BtGST2 was verified by obtaining a single band of the expected size in an SDS-PAGE gel (data not shown). The recombinant pure BtGST2 enzyme was catalytically active and substrate specificities for different model substrates (CDNB, DCNB, CuOOH) were determined. The specific activity of BtGST2 towards the model substrate CDNB (4.33 ± 0.64 μmol/min/mg) was low compared to that of other insect GSTs, which were previously associated with xenobiotic detoxification, such as the *Nl*GST1-1 from *Nilaparvata lugens*^31^, and the *Ag*GST1-5 from *Anopheles gambiae*^32^, but higher than that of *Tu*GSTd14 from *Tetranychus urticae*^33^ (Table 1). Specific activity towards DCNB (0.68 ± 0.28 μmol/min/mg) was comparable, within a range, to those reported for the aforementioned insect GSTs (Table 1). *Bt*GST2 displayed glutathione peroxidase activity against CuOOH (3.58 ± 0.08 μmol/min/mg), which was higher than the peroxidase activity reported for *Ag*GST1-5^32^ and *Tu*GSTd14^33^, and comparable to that of *Nl*GST1-1^31^ (Table 1).

The steady-state kinetic properties of *Bt*GST2 for the GSH/CDNB conjugation reaction were designated using various concentrations of both substrates. BtGST2 showed affinities K_m_ = 0.58 ± 0.06 mM and 0.71 ± 0.16 mM for GSH and CDNB, respectively, similar to that of NlGST1-1^31^, *Ag*GST1-5^32^, but higher than that of TuGSTd14^33^ (Table 2). In addition, for both GSH and CDNB, the k_*cat*_ values (8.71 and 9.75 min^−1^ for GSH and CDNB, respectively) and the k_*cat*_/K_m_ ratio values (15.01 and 13.73 mM^−1^ · min^−1^ for GSH and CDNB, respectively), were higher for *Bt*GST2 than the respective values reported for *Nl*GST1-1, *Ag*GST1-5 and *Tu*GSTd14 (Table 2), indicating an elevated catalytic activity and effectiveness for the GSH/CDNB conjugation reaction.

After initial enzymatic characterization, we determined the potential interaction (percentage inhibition of CDNB conjugation with GSH) of BtGST2, with six phytotoxins belonging to alkaloids (nicotine and caffeine), flavonoids (flavone and quercetin) and glucosinolates (I3C and AITC) groups (0.5 mM of each phytotoxin) (Table 3). Nicotine caused a 6.64 ± 3.68% inhibition while for caffeine, an inhibitory effect was not detected under experimental conditions, suggesting that BtGST2 may not be capable of interacting with these alkaloid moieties. On the other hand, the two flavonoids tested showed a strong inhibitory effect (flavone caused a 67.32 ± 8.65% while quercetin caused a 97.56 ± 2.43% reduction in CDNB/GSH conjugation by BtGST2). In a similar manner, BtGST2 was also found to likely interact with the isothiocyanates I3C and AITC, as these two compounds caused a 20.87 ± 7.99% and 40.93 ± 5.08% inhibition of CDNB conjugation, respectively. Collectively, these data suggest that BtGST2 may be involved in the detoxification of flavonoids and glucosinolates in *B. tabaci* but direct proof (GSH conjugation) is still lacking.

## Discussion

The principle of allocation, that organisms must partition limited resources among the processes of growth, reproduction and defense, has motivated most of the theory about the evolution of detoxification resistance to plant chemical defenses in generalists herbivores^34^,^35^. According to the theory, the inducible mode of detoxification resistance is assumed to be advantageous over the constitutive mode, because it allows to “turn on” the detoxification system only when required. This confers an adaptive phenotypic plasticity that enables reducing the costs associated with maintenance of metabolism and optimizing the organism’s fitness according to the levels of toxins in its environment^4^. In addition, it was suggested that inducibility may allow insects to tailor their detoxification profiles to specific mixture of multiple substrate toxins in their diets^36^.

In contrast, our analyses indicated that relatively few genes were induced in both the MEAM1 and MED species of *B. tabaci*, in response to the addition of phytotoxins to the sucrose diet. This finding complements extensive genomic surveys conducted in other insect systems which indicated that only a minority of detoxification genes respond to a variety of environmental inducers. Le Goff *et al.*^37^ used DNA microarray hybridization to examine P450 transcript accumulation patterns in response to two synthetic inducers (phenobarbital and atrazine) in *Drosophila melanogaster*. These authors found that only 11 out of 86 P450s were induced by phenobarbital and only seven by atrazine. In another dipteran, *Aedes aegypti*, Poupardin *et al.*^38^ examined the inducibility of genes that encode detoxification enzymes by a variety of environmental xenobiotics. They found a differential response, with only few of 168 P450 genes in the genome responding to any of the compounds tested. Recently, P450 induction profiles were measured in *Spodoptera frugiperda* larvae exposed to five plant secondary metabolites. Excluding xanthotoxin, which is known to be a potent inducer, only seven genes (from the 29 analyzed) were induced by indole, four by I3C, two by 2-tridecanone and none by quercetin^39^. It cannot be excluded at this stage that the simplified single-toxin diet utilized in all of these studies (including ours) contributed to the relatively limited induction of gene expression observed. To answer this, further research with more complex diets is required, testing for the possibility that additive or synergistic interactions among various constituents of the insect’s diet are required for efficient induction of its detoxification defense system.

Also, transcriptional regulation of detoxification in both specialist and generalist species appears to be relatively unspecialized. This phenomenon was exemplified in three different ways: one, lack of correlation between the probability of encountering a toxic compound and its inductive capabilities. In the generalist species *Helicoverpa zea*, synthetic substances like phenobarbital, and rare phytochemicals like xanthotoxin, were shown to be much more effective in up-regulating transcripts of detoxification enzymes than widespread phytochemicals like flavonoids^40^. Two, lack of correlation between inducibility levels of detoxification enzymes and resistance (i.e., the enzyme activity towards the inducing substrate). In *H. zea*, a detoxification enzyme with an overall lowest level of activity toward an array of substrates, showed the highest level of inducibility^13^. Three, inducibility was shown to lack the ability to function as a mechanism of tailoring detoxification rates to dietary intake. Even specialists, like the parsnip webworm *Depressaria pastinacella*, cannot adjust the pattern of detoxification to one particular furanocoumarin or to a mixture of furanocoumarins in their diet^36^. Moreover, given the diversity of detoxification enzymes within an insect genome and their multiplicity of functions (including the differential potential of bio-activation rather than detoxification), induction of large set of enzymes may actually enhance, rather than reduce, toxicity of specific plant compounds^41^. For example, P450 inducibility in the generalist *H. zea*, was detrimental in the presence of a plant pathogen that produces aflatoxin, a toxin that can be bio-activated by P450s activity^42^.

Together, these findings suggest that the importance of constitutive transcription of detoxification genes may have been previously underestimated. We hypothesize that for generalist herbivores, it may be more advantageous to move from one plant species to the other while constitutively expressing genes (coding for enzymes) capable of detoxifying a broad range of phytotoxins. This is mostly because these species are likely not to have the ability, anyway, to respond to specific phytotoxin with the “right” optimal gene. For example, the recent host shift to tobacco (*Nicotiana tabacum*) by the peach–potato aphid, *Myzus persicae*, was shown to be related to constitutive overexpression of a cytochrome P450 (CYP6CY3) which allows tobacco-adapted races of *M. persicae* to efficiently detoxify nicotine^43^. Again, it should be stated that it is also possible that efficient induction of the detoxification system in generalist insect herbivores requires the presence of phytotoxin mixtures and that the observed heavy reliance on constitutive expression can be at least partially related to the simplified diet we used.

When the constitutive transcription levels of the detoxification genes were compared between MEAM1 and MED adults feeding on sucrose diet lacking phytotoxins (‘sucrose only’), it was found that eight of the 18 genes were transcribed significantly higher in MED while only two genes were transcribed significantly higher in MEAM1 (Fig. 1b). In addition, the MED species showed a higher inducibility potential in response to the addition of phytotoxins to its sucrose diet (overall, nine induction events were recorded in MED while only four in MEAM1, Fig. 1a). Mahadav *et al.*^44^, using a heat-shock experimental system, also provided evidence supporting the existence of differences between the MEAM1 and MED regulation of stress-related genes as well as several genes encoding microfilament and cytoskeleton proteins which show significant higher levels of constitutive and induced transcription in MED than in MEAM1. The lower investment of MEAM1 in constitutive and induced resistance, can be beneficial to the fitness of this species at favorable conditions (feeding on sucrose diet for example) but the disadvantages of this strategy become apparent when the stress becomes acute (exemplified in this study by short period of feeding on a diet containing toxic indolyl glucosinolates). On the other hand, the high investment of MED in constitutive and induced defenses, likely introduces a ‘fitness-cost’ which reduces its reproductive performance both at favorable and acute stress conditions, but gives it the advantage of stability and security of equal performance in both environments^26^.

Although the “heat map” in Fig. 1a was dominated by yellow squares indicating no transcription differences within species in response to the presence of phytotoxins in the insect’s diet, our analyses identified three genes that responded to more than one compound. This includes the induction of *BtGST2* by I3C in the MED species, and by flavone and AITC in both species, the induction of *COE2* by caffeine and flavone in the MED species and the induction of *CYP6-like 5* by flavone in both species and by quercetin in the MED species. Moreover, all three genes were highly transcribed in the gut tissue (Fig. 3), and the enzyme coded by the *BtGST2* gene, showed significant interaction with various toxic phytotoxins (Table 3), such as I3C, AITC, flavone and quercetin that were capable of inducing the *BtGST2* gene (excluding quercetin). The idea the generalist insect species harbor enzymes capable of detoxifying a broad range of phytotoxins is well supported. In the generalist *H. zea*, the *CYP6B8* gene is induced by several plant phytotoxins such as xanthotoxin, I3C, flavone, gossypol, rutin, coumarin and quercetin^40^, and more importantly, the enzyme coded by the gene was shown to be capable of metabolizing most of the outlined inducers: xanthotoxin, flavone, rutin, I3C and quercetin^14^. Similarly, *CYP321A1* was induced in *H. zea* in response to the presence of several furanocoumarins and flavonoids^13^, and the enzyme was shown to metabolize xanthotoxin, angelicin (furanocoumarins) and α-naphthoflavone^45^,^46^. In a similar manner, although documented primarily at the biochemical level, GST isoenzymes isolated from the polyphagous fall armyworm, *S. frugiperda*, were shown to be induced and capable of metabolizing α,β-unsaturated carbonyls^47^ and organo-thiocyanates^48^.

How a broad range of structurally and biochemically un-related phytotoxins induces generalist detoxification genes remains largely unknown. To date, only few reports have successfully underlined the molecular mechanism of induced detoxification response to plant toxins in insect herbivores. Comparison of upstream regulatory sequences of the specialist *Papilio polyxenes CYP6B1* gene and the generalist *Papilio glaucus CYP6B4* gene, revealed the involvement of conserved transcription-regulatory elements in the regulation of constitutive transcription of these genes and their induction by furanocoumarins^47^. Variation in the overlapping EcRE (ecdysone-response element)/ARE (antioxidant-response element)/XRE (xenobiotic-response element)-xan sequence and a xenobiotic response element to aryl hydrocarbon receptor (XRE-AhR), including the additional EcRE and octamer 1 (Oct-1) elements in the generalist promoter and, probably most importantly, their more extended spacing, was assumed to account for the low constitutive transcription of the generalist promoter and its high inducibility^49^,^50^. More recently, an element, designated as xenobiotic response element to flavone (XRE-Fla), containing a 5′ AT-only TAAT inverted repeat, a GCT mirror repeat and a 3′ antioxidant response like element, was identified in the *H. zea CYP321A1* promoter region. Electrophoresis mobility shift assays demonstrated that XRE-Fla, specifically binds to *H. zea* fatbody cell nuclear extracts and that flavone treatment increases the nuclear concentrations of uncharacterized transcription factors capable of binding to XRE-Fla^51^. Interestingly, the XRE-Fla region also serves as an essential element for xanthotoxin-dependent promoter induction^52^. In *M. persicae*, the aforementioned constitutive overexpression of CYP6CY3 in tobacco-adapted races was found to result from expansion of a dinucleotide microsatellite (AC(n)) in the promoter region (from 15 repeat units in *M. persicae sensu stricto* clones to 48 repeat units in *M. persicae nicotianae* clones) and a recent gene amplification, with some aphid clones carrying up to 100 copies^43^.

As indicated in Fig. 1a, the transcription of several *CYP* genes can also be decreased after 24 h of exposure to some phytotoxins. However, compared with our knowledge of P450 induction, the mechanisms involved in P450 suppression in insects are poorly understood. We can only speculate here that decreases in *CYP* gene transcription could be an adaptive or homeostatic response to protect the cell from the deleterious effects of the modified/bio-activated molecule, the P450 derived oxidizing species, and/or a need for the tissue to utilize its transcriptional machinery and energy for the synthesis of other components involved in the defense response. In mammals, the following general themes were suggested to characterize P450 suppression: I) diverse chemical signals can trigger P450 suppression; II) multiple molecular mechanisms can be involved in transcriptional suppression. III) the processes often involve rather complex cascades of transcription factors and other regulatory proteins^53^.

The *B. tabaci* species complex is recognized to be one of the worst, all-time, global pests^19^, threatening food security in the developing world, including Africa, the Asian Pacific, and South America. Still, at the molecular level, practically nothing is known on the mechanism/s involved in this insect’s tremendous ability to successfully utilize hundreds of plant species as hosts and in many cases to establish super-abundant populations. Our results provide a first insight into the correlation between detoxification gene transcription and the insect response to the presence of specific phytotoxins in its diet. They also highlight the urgent need to combine the obtained transcriptomic data with metabolomics, gene-silencing and *in vitro* expression approaches for gaining a thorough basic understanding of the insect’s fundamental biology related to plant-insect interactions that will also enhance the development of new crop defense strategies.

## Methods

### Origin and maintenance of insect strains

Two *B. tabac*i colonies, collected from fields in Israel, were used in this study: B-ref (collected from cotton fields in the Ayalon Valley in 1987) and PyriR-unsel (collected in 1991 from a rose greenhouse in the southwest of Israel). Based on their cytochrome oxidase I (COI) sequences, B-ref was designated as the MEAM1 (B) species while PyriR-unsel was designated as the MED (Q) species. Since their collection, the MEAM1 and MED colonies were maintained in separate rooms, and reared under standard greenhouse conditions of 26 ± 2 °C, photoperiod of L:D 14 h:10 h on cotton (*Gossypium hirsutum* L cv Acala). The homogeneity and purity of the colonies was verified every several generations by cleaved amplified polymorphic sequences (CAPS) of the COI gene^23^.

### Artificial feeding assays

Feeding assays were performed for 24 h on 10% sucrose solution containing either 0.0015% nicotine (alkaloid), 0.005% caffeine (alkaloid), 0.1% flavone (2-phenyl chromone), 0.1% quercetin (a flavonolaglycone), 0.02% indole-3-carbinol (I3C), the breakdown product of indol-3-ylmethylglucosinolate and 0.001% allyl-isothiocyanate (AITC), the hydrolysis product of the aliphatic glucosinolate sinigrin (Sigma). These phytotoxins were chosen to represent a range of probable ecological encounter rates. The flavonoids flavone and quercitin are present in a wide range of *B. tabaci* host families. Flavonoids are translocated in the phloem^54^, and were tightly associated with resistance to aphids^55^. Moreover, analyses of cassava phloem sap and honeydew excretion samples of the phloem feeding mealybug *Phenacoccus manihoti* indicated both ingestion and metabolic processing of flavonoids by the insect^54^. Nicotine is a solanaceous phytotoxin, a host plant family commonly utilized by *B. tabaci*. Nicotine is synthesized in roots and transported via the xylem to foliar tissues in tobacco^56^. Nicotine levels in tobacco phloem sap have not been quantified. However, tobacco-feeding phloem feeders are likely to also come into contact with nicotine through periodic ingestion of xylem sap and through exposure to alkaloid secretions by glandular trichomes^57^. Glucosinolates and their breakdown products occur primarily in Brassicaceae, a family that is occasionally utilized by *B. tabaci*. The transport of glucosinolates in plants occurs from source to sink by diffusion in the phloem^58^. Caffeine is encountered in six primarily caffeine-containing genera: *Camellia*, *Coffea*, *Cola*, *Ilex*, *Paullinia* and *Theobroma*. These genera are rarely utilized as host plants by *B. tabaci*. Caffeine was found in phloem exudates of coffee (*Coffea arabica*) seedlings^59^.

To our knowledge, reliable measurement of concentrations for different phytotoxins in the plant phloem is unreliable due to contaminants from the surrounding tissues. Hence, we chose sub-lethal concentrations that did not kill the feeding insects that should better reflect the response to a specific rather than a general stress response in dying insects. We performed preliminary experiments to determine accurate sub-lethal concentration for each compound, meaning that we looked for the highest concentration in which mortality did not differ between ‘sucrose only’ and ‘sucrose + phytotoxin’ diets.

For dissolution of flavone and quercetin, 1% acetone was added. Control adults were fed for 24 h with a 10% sucrose solution lacking any additives apart from the dissolving agents (‘sucrose only’ diet). The feeding system contained a plastic tube (5 cm high × 2.5 cm diameter) with the liquid diet contained within a double layer of Parafilm. 100 newly emerged adults were placed in each plastic tube. Surviving individuals were collected and counted at the end of the feeding period.

### Detoxification genes transcription in the MEAM1 and MED species of *B. tabaci*

In order to identify detoxification genes, in MEAM1 and MED, that are induced by the presence of phytotoxins in the insect’s diet, RNA was extracted from a pool of about 50 surviving individuals. One μg of total RNA were treated with DNase (Promega), and the RNA was re-extracted in phenol: chloroform followed by an ethanol precipitation. First strand cDNA was synthesized by Superscript II (Invitrogen) using 1 μg total RNA and an Oligo-dT primer according to the manufacturer’s instructions.

Quantitative real-time PCR (qRT-PCR) analyses focused on a set of eighteen detoxification genes, believed to play a key role in *B. tabaci* detoxification responses to environmental stress, due to their recurrent appearance in our previously reported cDNA libraries of strains showing susceptibility or resistance to several groups of chemical insecticides and to flavonoids^27^,^28^. These include: 12 P-450s, from the *CYP4* and *CYP6* families, three GSTs from the sigma and omega families, two type-B COEs and one UDP-GT (list of names, accession numbers, RT-PCR primers and product sizes are provided in Table S3 and the Minimum Information for Publication of Quantitative Real-Time PCR Experiments [MIQE] is provided in Table S4). qRT-PCR reactions were performed using GeneAmp 7300 (Applied Biosystems). The PCR conditions, including the cDNA dilutions and primer concentrations, were adjusted for the amplification efficiencies of all genes to be similar, and were optimally set to master mix (18μl) containing: 9 μl Absolute QPCR SYBR Green Mix (ABgene), 150 nM or 250 nM forward and reverse primers and 2 μl of cDNA. PCR thermal conditions consisted one cycle of 50 °C for 2 min, one cycle of 95 °C for 2 min, followed by 40 cycles of 95 °C for 15 sec and 60 °C for 1 min. Melting curves were conducted for all samples to check for specific gene amplification (single peak). Moreover, no template controls (no cDNA in PCR) were run for each gene to detect unspecific amplification and primer dimerization. When present, the signal amplification plot was very late (Cq > 34) and there was a high difference between the negative control and all the cDNA samples. Primer efficiencies were ≥ 0.91 and ≤ 1, excluding one gene (*Cyp6EE600001* = 0.87). Quantification of the transcript level was conducted according to the ∆CT method using actin (accession number KC16211) as the reference gene (see justification below). Each reaction was performed in triplicate to minimize intra-experiments variation, and the mean of three independent biological replicates (three different cDNA samples) was calculated. Gene induction data were analyzed by one-way ANOVA model (diet-type, ‘sucrose only’ versus sucrose plus phytotoxin, as the main effect) for each species, gene and phytotoxin separately. Genes were considered significantly over- or under-transcribed when the ∆CT values of RNA samples from sucrose plus phytotoxin diet were different from ∆CT values of RNA samples from ‘sucrose only’ diet at *P* ≤ 0.05. Comparisons of constitutive transcription levels of detoxification genes (transcription levels when feeding on ‘sucrose only’ diet) were conducted by one-way ANOVA model (species, MEAM1 versus MED, as the main effect) for each gene separately. Genes were considered significantly over- or under-transcribed when the ∆CT values of RNA samples from MED were different from ∆CT values of RNA samples from MEAM1 at *P* ≤ 0.05. All statistical analyses conducted in this paper (see below) were performed with JMP statistical software version 10 (SAS Institute, USA).

We found four papers published after we initiated our experiments that discuss the use of qRT-PCR reference genes in *B. tabaci* (papers are listed in the relevant section of the MIQE table). While these papers do not recommend actin (accession number KC16211) as the best reference gene for *B. tabaci*, overview of the insights of the four papers raised the understanding that (i) each paper came with different and sometimes contradicting recommendations (ii) major changes in ranking occurred frequently and different genes were recommended for different treatments within a single paper (iii) different optimization programs can reach different conclusion and therefore (iv) each experimental system should be carefully tailored to answer the questions asked. Here, we base our argument that actin was a proper choice as an individual reference gene in our experimental system using two independent findings: (1) evaluation of the gene stability in our experimental system and (2) general statistical analysis of actin expression in our experimental system.

(1) We analyzed the stability of actin and five other genes in our experimental system (*Cyp4-like 2*, *Cyp6-like 3*, *Cyp6CM1*, *UDP-GT* and *BtGST3*) which did not respond to any of the reported phytotoxins treatments (Fig. 1a), using the programs Normfinder (http://moma.dk/normfinder-software) and geNorm (https://genorm.cmgg.be/). In Normfinder, the stability value was 0.33 and was ranked first together with *Cyp6-like 3* (same stability value). *BtGST3* was ranked third with stability value of 0.52. The Normfinder stability value of 0.33 was comparable to that of recommended reference genes in the aforementioned *B. tabaci* papers. Moreover, using the pair *Cyp6-like 3* and actin only reduces the stability value to 0.27, suggesting that adding an additional reference gene will not significantly improve the analysis. In the geNorm analysis, the M stability values of actin (step 1) ranged between 0.012–0.0720 which was below 0.15 and therefore indicated that an additional reference gene will not significantly improve normalization.

(2) we also conducted two-way ANOVA analysis on the obtained Ct values of actin using: “treatment” (‘sucrose only’ and ‘sucrose + phytotoxin’) and “phytotoxin” (‘quercetin’, ‘AITC’, ‘flavone’, ‘I3C’, ‘caffeine’ and ‘nicotine’) as the main effects. Main effect comparison between the Ct means of ‘sucrose only’ (17.78 ± 1.1 [SD]) and ‘sucrose + phytotoxin’ (17.68 ± 1.32 [SD]) indicated non-significant difference (*P* = 0.21). Moreover, the same pattern was obtained when the two treatments were compared within each compound separately (data not shown).

### Oviposition, egg development and survival

Oviposition, development and survival experiments were conducted in a temperature-controlled room in long day conditions (14 h L/10 h D, 24 ± 1 °C). Fifty *B. tabaci* couples from MEAM1 and MED, where fed on an artificial diet of 10% sucrose containing 0.02% I3C or 10% ‘sucrose only’ diet for 24 h. Then, individual females were placed in 2.5-cm Petri dishes, with 1 cm hole covered with 50 mash net, containing cotton leaf discs. All discs were placed with their adaxial surface downwards onto a bed of 1% Bacto-agar (Becton, Dickinson & company). The number of eggs oviposited by each female was monitored at 24, 48, 72 and 96 h. At each time point, the position of every egg was marked on a paper image of the cotton leaf. This method allowed us to follow the development time to first instar nymph and viability of each egg separately. After 4 days (from artificial diet feeding completion), ovipositing females were removed and eggs development was monitored for additional 8 days. Each (species X feeding treatment) combination was repeated 15 times. Eggs that did not hatch 8 days after oviposition were scored as dead. Differences in oviposition rate (eggs/female/day), egg developmental period (days) and survival were tested for significance by a two-way ANOVA model, in which species and artificial diet types were set as fixed effects. Specific means in the (species X feeding treatment) interaction were selected *a priori* and orthogonal comparisons were conducted. Statistical significance was assumed at *P* ≤ 0.05.

### Gene transcription localization

To test for gut specific transcription, total RNA was extracted from gut and the rest of the body (excluding gut) tissues of 50 females (each biological sample). Then, all samples were subjected to first-strand cDNA synthesis and qRT-PCR analyses as described above. Each reaction was performed in triplicate to minimize intra-experiments variation, and the mean of at least three independent biological replicates was calculated. Comparisons of transcription levels between gut and the rest of the body samples were conducted by one-way ANOVA model for each gene separately. As described above, genes were considered significantly over- or under-transcribed when the ∆CT values of RNA samples from the gut were different from ∆CT values of RNA samples from the rest of the body at *P* ≤ 0.05.

### BtGST2 expression and inhibition assays

Sequence encoding for BtGST2 was amplified from MED cDNA using 5′-CACCATGGCTCCTCCCAAGCTGACGTAC-3′ and 5′-TCACCAGTCGGTTACCGG CCTTTTG-3′ primers using a Pfu polymerase (Thermo Scientific). PCR conditions were 95 °C for 3 min, followed by 35 cycles of 95 °C for 30 sec, 61 °C for 30 sec and 60 °C for 30 sec. The PCR product was gel purified using Nucleospin Extract II kit (Macherey-Nagel) and cloned into pET100/D-TOPO vector (Invitrogen Life Technology). Plasmid extraction was performed using Nucleospin Plasmid kit (Macherey-Nagel) and a construct of the correct DNA sequence was selected for protein expression. *Escherichia coli* BL21 (DE3) competent cells were transformed with the construct and a single colony was grown in 2 liters of Luria-Bertani (LB) medium in the presence of 100 μg/ml ampicillin at 37 °C. When absorbance at 590 nm was 0.9, the expression of BtGST2 was induced by the addition of Isopropyl β-D-1-thiogalactopyranoside (IPTG) to a final concentration of 1 mM. The culture was further grown at 37 °C for four hours and after that, cultures were centrifuged at 5,000 g for 20 min, cell pellet was re-suspended in 25 ml of 160 mM sodium phosphate buffer pH 7.4. Cells were lysed by multiple rounds of freeze/thawing followed by incubation with 10 μg/ml of lysozyme (Sigma-Aldrich) on ice for 30 min and sonication. The lysate was centrifuged at 10,000 g for 30 min at 4 °C to remove the cell debris and the supernatant was loaded to a pre-equilibrated Ni-NTA agarose column (Qiagen). Unbound proteins were washed off with 15 volumes of 160 mM sodium phosphate buffer pH 7.4 and the BtGST2 was eluted using 160 mM sodium phosphate buffer pH 7.4 containing 500 mM imidazole. The eluate was cleaned-up from small molecules as imidazole via PD-10 size exclusion column (GE Healthcare) following manufacturer’s instructions. Protein was dialyzed in 50 mM phosphate buffer pH 7.4 and glycerol (40% final concentration) was added for long-term storage.

Glutathione peroxidase activity against cumene hydroperoxide (CuOOH) was measured indirectly at 340 nm at 25 °C, by a coupled reaction of glutathione reductase according to the method described by Simmons *et al.*^60^. Activity against 1-chloro-2, 4dinitrobenzene (CDNB) and 1,2-dicloro-4-nitrobenzene (DCNB) was measured at 25 °C based on a published method. Briefly, the measurements were performed in 0.1 M potassium phosphate buffer pH 6.5 using 2.75 mM glutathione (GSH) and 0.99 mM CDNB or DCNB, respectively. The formation of the corresponding conjugate of CDNB or DCNB with GSH was monitored at 340 nm for 2 min. For the determination of kinetic parameters (K_m_, k_cat_), measurements were performed by varying the concentration of GSH (0.15–6 mM) and keeping the concentration of CDNB constant (0.99 mM) and vice versa, by varying the concentration of CDNB (0.06–3 mM) and keeping the concentration of GSH constant (2.75 mM). K_m_ and k_cat_ values were determined by fitting the resulting data to the Michaelis-Menten equation in GraFit 3 software (Ericathus Software Ltd.). The interaction of BtGST2 with selected phytotoxins (nicotine, caffeine, flavone, quercetin, I3C, AITC; Sigma-Aldrich) was studied by monitoring the inhibition of CDNB conjugating activity in the presence of 0.5 mM of each phytotoxin. The reactions were performed in a 10% organic solvent (methanol) in order to increase the solubility of phytotoxins after confirmation that methanol did not influence the enzyme activity. All measurements were performed in triplicates in 96-well plates (NuncMaxiSorp) using a Spectra Max M2e multimode microplate reader (Molecular Devices, Berkshire, UK).

## Additional Information

**How to cite this article**: Halon, E. *et al.* Only a minority of broad-range detoxification genes respond to a variety of phytotoxins in generalist **Bemisia tabaci** species. *Sci. Rep.* **5**, 17975; doi: 10.1038/srep17975 (2015).

## Supplementary Material

Supplementary Information

## Figures and Tables

**Table 1:** Specific activities of *Bt*GST2 and other insect and mite GSTs previously associated with xenobiotics detoxification, towards selected model substrates.

Substrate	Specific activity^a^ (Unit^b^/mg)			
	**BtGST2**	**NlGST1-1**^c^,^d^	**AgGST1-5**^c^,^e^	**TuGSTd14**^c^,^f^
1-Chloro-2,4-dinitrobenzene (CDNB)	4.33 ± 0.64	141 ± 16.4	56.4 ± 8.7	0.69 ± 0.05
1,2-Dichloro-4-nitrobenzene (DCNB)	0.68 ± 0.28	0.9 ± 0.12	0.32 ± 0.03	0.08 ± 0.01
Cumene hydroperoxide (CuOOH)	3.58 ± 0.08	5.10 ± 0.41	< 0.13 ± 0.01	0.10 ± 0.01

^a^The amount of product produced per minute per milligram of total enzyme at 25 °C.

^b^One unit of enzyme catalyzes the reaction of 1 μmol of substrate per minute at 25 °C. Data are means of three replicates ± S.D.

^c^Nl = *Nilaparvata lugens*; Ag = *Anopheles gambiae*; Tu = *Tetranychus urticae*.

^d^Data from Vontas *et al.*^31^.

^e^Data from Ranson *et al.*^32^.

^f^Data from Pavlidi *et al.*^33^.

**Table 2:** Kinetic parameters of *Bt*GST2 for the GSH/CDNB conjugation reaction, compared to other insect and mite GSTs previously associated with xenobiotics detoxification.

Kinetic para meter	BtGST2	NlGST1-1^a^,^b^	AgGST1-5^a^,^c^	TuGSTd24^a^,^d^
Km for GSH (mM)	0.58 ± 0.06	0.65 ± 0.07	0.82	3.79 ± 0.69
Km for CDNB (mM)	0.71 ± 0.16	0.26 ± 0.02	0.09	1.69 ± 0.24
k*cat* (min^−1^) (GSH)	8.71 ± 0.29	1.96	0.66	1.22 ± 0.10
k*cat* (min^−1^) (CDNB)	9.75 ± 0.88	—	0.61	1.78 ± 0.25
K*cat*/Km (mM^−1^· min^−1^) (GSH)	15.01	1.27	0.80	0.32
K*cat*/Km (mM^−1^· min^−1^) (CDNB)	13.73	—	6.77	1.08

^a^Nl = *Nilaparvata lugens*; Ag = *Anopheles gambiae*, Tu = *Tetranychus urticae*.

^b^Data from Vontas *et al.*^31^.

^c^Data from Ranson *et al.*^32^.

^d^Data from Pavlidi *et al.*^33^.

**Table 3:**
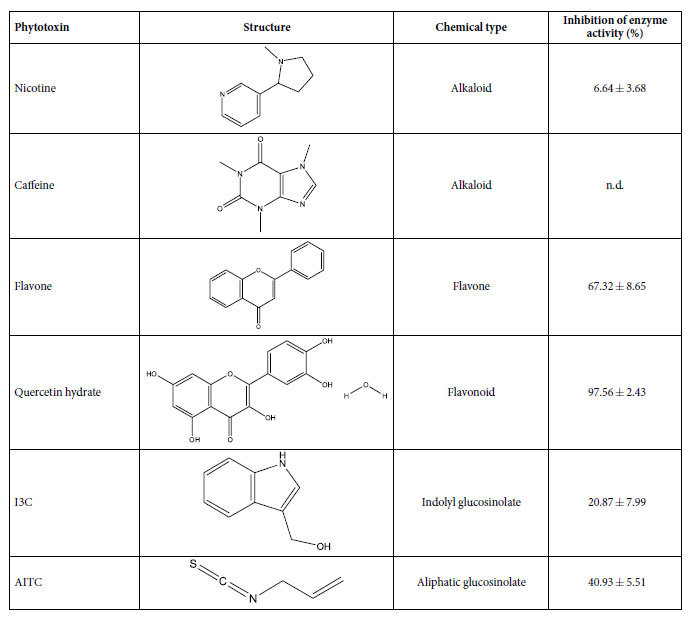
Percentage inhibition of CDNB conjugation with GSH by BtGST2, using six phytotoxins belonging to alkaloids (nicotine and caffeine), flavonoids (flavone and quercetin) and glucosinolates (I3C and AITC) groups.

Data are mean of three replicates ± S.D. BtGST2 was assayed using 2.75 mM GSH and 0.99 mM CDNB as substrates and 0.5 mM of each phytotoxin. n.d.: not detected under assay conditions.
