# Supplementary material for: Only a minority of broad-range detoxification genes respond to a variety of phytotoxins in generalist Bemisia tabaci species
Source: Sci Rep. 2015 Dec 10;5:17975. doi: 10.1038/srep17975 (PMC4674796; doi:10.1038/srep17975)
Supplement: Supplementary Information [file srep17975-s1.pdf]

Supplementary information

Only a minority of broad-range detoxification genes respond to a variety of phytotoxins in generalist *Bemisia tabaci* species

Eyal Halon, Galit Eakteiman, Pnina Moshitzky, Moshe Elbaz, Michal Alon, Nena Pavlidi, John Vontas & Shai Morin

Table S1 Detailed ΔΔCT values and statistics of the ΔCT method, for each species, gene and phytotoxin separately. Genes were considered significantly over- or under-transcribed when the ΔCT values of RNA samples from sucrose plus phytotoxin diet (S + P) were different from ΔCT values of RNA samples from 'sucrose only' diet (S) at $P \leq 0.05$.

Caffeine

Gene	Value	MEAM1 (S+P) / (S)	MED (S+P) / (S)
COE1	t=	-0.572	0.7106
	$2^{-\Delta\Delta CT}$ estimates	1.379	0.67
	p(t)	0.5831	0.4975
COE2	t=	0.2624	-2.557
	$2^{-\Delta\Delta CT}$ estimates	0.942	1.788
	p(t)	0.7996	0.0338
Cyp4-like 1	t=	-1.377	-1.254
	$2^{-\Delta\Delta CT}$ estimates	1.96	1.847
	p(t)	0.2057	0.2454
Cyp4-like2	t=	0.4278	-1.307
	$2^{-\Delta\Delta CT}$ estimates	0.823	1.811
	p(t)	0.6801	0.2276
Cyp4-like 4	t=	1.7626	-1.303
	$2^{-\Delta\Delta CT}$ estimates	0.61	1.442
	p(t)	0.116	0.2289
Cyp4-like 5	t=	0.4328	-0.762
	$2^{-\Delta\Delta CT}$ estimates	0.884	1.242
	p(t)	0.6766	0.4677

Cyp6-like 1	t=	1.1033	0.4573
	$2^{-\Delta\Delta CT}$ estimates	0.762	0.894
	p(t)	0.302	0.6596
Cyp6-like 2	t=	0.4867	-0.494
	$2^{-\Delta\Delta CT}$ estimates	0.837	1.2
	p(t)	0.6395	0.6348
Cyp6-like 3	t=	-1.128	-0.378
	$2^{-\Delta\Delta CT}$ estimates	1.278	1.086
	p(t)	0.2921	0.7155
Cyp6-like 4	t=	-3.709	0.1641
	$2^{-\Delta\Delta CT}$ estimates	2.576	0.959
	p(t)	0.006	0.8737
Cyp6-like 5	t=	-1.777	-0.218
	$2^{-\Delta\Delta CT}$ estimates	1.636	1.062
	p(t)	0.1136	0.8332
Cyp6CM1	t=	-0.228	-0.676
	$2^{-\Delta\Delta CT}$ estimates	1.094	1.305
	p(t)	0.8257	0.5184
Cyp6-like EE600001	t=	-0.126	0.5733
	$2^{-\Delta\Delta CT}$ estimates	1.048	0.808
	p(t)	0.903	0.5822
BtGST1	t=	-0.58	-0.75
	$2^{-\Delta\Delta CT}$ estimates	1.139	1.183
	p(t)	0.5778	0.4747

BtGST2	t=	-1.515	1.7542
	$2^{-\Delta\Delta CT}$ estimates	1.311	0.73
	p(t)	0.1682	0.115
BtGST3	t=	0.0142	0.3074
	$2^{-\Delta\Delta CT}$ estimates	0.999	0.975
	p(t)	0.989	0.7664
Cyp6-like	t=	-0.808	-0.171
	$2^{-\Delta\Delta CT}$ estimates	1.29	1.056
	p(t)	0.4455	0.8687
UDP-GT	t=	-1.01	1.9173
	$2^{-\Delta\Delta CT}$ estimates	1.521	0.451
	p(t)	0.3423	0.0915

Flavone

Gene	Value	MEAM1 (S+P) / (S)	MED (S+P) / (S)
COE1	t=	1.7859	1.5956
	$2^{-\Delta\Delta CT}$ estimates	0.645	0.676
	p(t)	0.1119	0.1492
COE2	t=	-0.863	-3.439
	$2^{-\Delta\Delta CT}$ estimates	1.241	2.369
	p(t)	0.4134	0.0088
Cyp4-like 1	t=	-1.907	-0.912
	$2^{-\Delta\Delta CT}$ estimates	2	1.393

	p(t)	0.0929	0.3884
Cyp4-like2	t=	-1.294	0.001
	$2^{-\Delta\Delta CT}$ estimates	1.856	1
	p(t)	0.2318	0.9993
Cyp4-like 4	t=	2.0679	1.5927
	$2^{-\Delta\Delta CT}$ estimates	0.524	0.608
	p(t)	0.0725	0.1499
Cyp4-like 5	t=	0.0733	0.1571
	$2^{-\Delta\Delta CT}$ estimates	0.967	0.93
	p(t)	0.9433	0.879
Cyp6-like 1	t=	0.3948	-1.818
	$2^{-\Delta\Delta CT}$ estimates	0.827	2.403
	p(t)	0.7033	0.1066
Cyp6-like 2	t=	4.1467	0.1249
	$2^{-\Delta\Delta CT}$ estimates	0.321	0.966
	p(t)	0.0032	0.9037
Cyp6-like 3	t=	0.7773	-1.051
	$2^{-\Delta\Delta CT}$ estimates	0.733	1.522
	p(t)	0.4594	0.3241
Cyp6-like 4	t=	1.1399	0.0816
	$2^{-\Delta\Delta CT}$ estimates	0.688	0.974
	p(t)	0.2873	0.937
Cyp6-like 5	t=	-4.056	-3.078
	$2^{-\Delta\Delta CT}$ estimates	3.074	2.344

	p(t)	0.0037	0.0152
Cyp6CM1	t=	-0.163	-1.832
	$2^{-\Delta\Delta CT}$ estimates	1.075	2.256
	p(t)	0.8747	0.1043
Cyp6-like EE600001	t=	6.0306	2.6534
	$2^{-\Delta\Delta CT}$ estimates	0.264	0.557
	p(t)	0.0003	0.0291
BtGST1	t=	0.1853	-1.008
	$2^{-\Delta\Delta CT}$ estimates	0.908	1.693
	p(t)	0.8576	0.3429
BtGST2	t=	-2.215	-3.352
	$2^{-\Delta\Delta CT}$ estimates	3.274	6.013
	p(t)	0.0576	0.01
BtGST3	t=	1.6726	0.3496
	$2^{-\Delta\Delta CT}$ estimates	0.544	0.88
	p(t)	0.1329	0.7356
Cyp6-like	t=	-0.04	-1.816
	$2^{-\Delta\Delta CT}$ estimates	1.01	1.609
	p(t)	0.9693	0.1068
UDP-GT	t=	1.5353	0.0701
	$2^{-\Delta\Delta CT}$ estimates	0.503	0.969
	p(t)	0.1633	0.9458

Indole 3 Carbinol (I3C)

Gene	Value	MEAM1 (S+P) / (S)	MED (S+P) / (S)
COE1	t=	1.8258	2.7713
	$2^{-\Delta\Delta CT}$ estimates	0.674	0.549
	p(t)	0.1053	0.0242
COE2	t=	0.259	-0.349
	$2^{-\Delta\Delta CT}$ estimates	0.884	1.181
	p(t)	0.8022	0.7358
Cyp4-like 1	t=	-0.907	-4.597
	$2^{-\Delta\Delta CT}$ estimates	1.407	5.66
	p(t)	0.3911	0.0018
Cyp4-like2	t=	0.206	-0.65
	$2^{-\Delta\Delta CT}$ estimates	0.896	1.411
	p(t)	0.8419	0.5342
Cyp4-like 4	t=	3.683	2.274
	$2^{-\Delta\Delta CT}$ estimates	0.505	0.656
	p(t)	0.0062	0.0526
Cyp4-like 5	t=	0.2306	0.418
	$2^{-\Delta\Delta CT}$ estimates	0.92	0.859
	p(t)	0.8234	0.687
Cyp6-like 1	t=	1.2761	-0.286
	$2^{-\Delta\Delta CT}$ estimates	0.493	1.171
	p(t)	0.2377	0.7822
Cyp6-like 2	t=	2.7818	0.8691
	$2^{-\Delta\Delta CT}$ estimates	0.353	0.722

	p(t)	0.0239	0.4101
Cyp6-like 3	t=	1.5217	-0.963
	$2^{-\Delta\Delta CT}$ estimates	0.699	1.254
	p(t)	0.1666	0.3637
Cyp6-like 4	t=	0.5985	-0.392
	$2^{-\Delta\Delta CT}$ estimates	0.723	1.237
	p(t)	0.5661	0.705
Cyp6-like 5	t=	1.1647	-0.921
	$2^{-\Delta\Delta CT}$ estimates	0.706	1.317
	p(t)	0.2777	0.3842
Cyp6CM1	t=	1.1262	-1.955
	$2^{-\Delta\Delta CT}$ estimates	0.66	2.059
	p(t)	0.2927	0.0863
Cyp6-like EE600001	t=	2.3632	0.4415
	$2^{-\Delta\Delta CT}$ estimates	0.459	0.865
	p(t)	0.0457	0.6706
BtGST1	t=	0.4753	-1.572
	$2^{-\Delta\Delta CT}$ estimates	0.851	1.708
	p(t)	0.6473	0.1546
BtGST2	t=	-0.035	-4.029
	$2^{-\Delta\Delta CT}$ estimates	1.173	6.126
	p(t)	0.9726	0.0038
BtGST3	t=	1.6282	2.0407
	$2^{-\Delta\Delta CT}$ estimates	0.618	0.548

	p(t)	0.1421	0.0756
Cyp6-like	t=	0.0246	2.3905
	$2^{-\Delta\Delta CT}$ estimates	0.994	0.558
	p(t)	0.981	0.0438
UDP-GT	t=	2.033	0.9578
	$2^{-\Delta\Delta CT}$ estimates	0.413	0.659
	p(t)	0.0765	0.3662

Allyl isothiocyanate (AITC)

Gene	Value	MEAM1 (S+P) / (S)	MED (S+P) / (S)
COE1	t=	1.8965	1.6372
	$2^{-\Delta\Delta CT}$ estimates	0.625	0.667
	p(t)	0.0945	0.1402
COE2	t=	0.9014	-1.338
	$2^{-\Delta\Delta CT}$ estimates	0.813	1.36
	p(t)	0.3937	0.2178
Cyp4-like 1	t=	-0.874	-0.513
	$2^{-\Delta\Delta CT}$ estimates	1.259	1.145
	p(t)	0.4075	0.6217
Cyp4-like2	t=	0.7091	0.3133
	$2^{-\Delta\Delta CT}$ estimates	0.744	0.877
	p(t)	0.4984	0.7621
Cyp4-like 4	t=	1.9631	0.6506

	$2^{-\Delta\Delta CT}$ estimates	0.728	0.9
	p(t)	0.0852	0.5336
Cyp4-like 5	t=	0.2619	3.1974
	$2^{-\Delta\Delta CT}$ estimates	0.962	0.621
	p(t)	0.8	0.0127
Cyp6-like 1	t=	-0.245	1.8397
	$2^{-\Delta\Delta CT}$ estimates	1.047	0.709
	p(t)	0.8123	0.1031
Cyp6-like 2	t=	0.7266	2.2667
	$2^{-\Delta\Delta CT}$ estimates	0.835	0.571
	p(t)	0.4882	0.0532
Cyp6-like 3	t=	0.6171	0.216
	$2^{-\Delta\Delta CT}$ estimates	0.8746	0.954
	p(t)	0.5543	0.8344
Cyp6-like 4	t=	1.0026	0.6225
	$2^{-\Delta\Delta CT}$ estimates	0.742	0.831
	p(t)	0.3454	0.5509
Cyp6-like 5	t=	-0.127	0.4597
	$2^{-\Delta\Delta CT}$ estimates	1.035	0.883
	p(t)	0.9023	0.658
Cyp6CM1	t=	0.3589	0.9235
	$2^{-\Delta\Delta CT}$ estimates	0.891	0.743
	p(t)	0.729	0.3828
Cyp6-like EE600001	t=	1.4029	1.8926

	$2^{-\Delta\Delta CT}$ estimates	0.626	0.531
	p(t)	0.1983	0.095
BtGST1	t=	-1.504	-0.772
	$2^{-\Delta\Delta CT}$ estimates	1.31	1.149
	p(t)	0.1709	0.4622
BtGST2	t=	-9.133	-7.78
	$2^{-\Delta\Delta CT}$ estimates	3.948	3.22
	p(t)	1.70E-05	0.0001
BtGST3	t=	-1.505	-0.49
	$2^{-\Delta\Delta CT}$ estimates	1.56	1.156
	p(t)	0.1707	0.6373
Cyp6-like	t=	-0.04	0.8618
	$2^{-\Delta\Delta CT}$ estimates	1.007	0.848
	p(t)	0.5553	0.4139
UDP-GT	t=	-0.791	1.0853
	$2^{-\Delta\Delta CT}$ estimates	1.221	0.76
	p(t)	0.452	0.3094

Nicotine

**Gene**	**Value**	**MEAM1 (S+P) / (S)**	**MED (S+P) / (S)**
COE1	t=	1.1749	-0.987
	$2^{-\Delta\Delta CT}$ estimates	0.725	1.3
	p(t)	0.2738	0.3528

COE2	t=	0.7175	-0.148
	$2^{-\Delta\Delta CT}$ estimates	0.748	1.061
	p(t)	0.4935	0.886
Cyp4-like 1	t=	0.986	-1.677
	$2^{-\Delta\Delta CT}$ estimates	0.604	2.355
	p(t)	0.353	0.132
Cyp4-like2	t=	1.6515	-1.107
	$2^{-\Delta\Delta CT}$ estimates	0.434	1.75
	p(t)	0.1372	0.3003
Cyp4-like 4	t=	-0.526	-1.439
	$2^{-\Delta\Delta CT}$ estimates	1.215	1.704
	p(t)	0.6134	0.1882
Cyp4-like 5	t=	-1.406	0.6918
	$2^{-\Delta\Delta CT}$ estimates	2.013	0.709
	p(t)	0.1978	0.5086
Cyp6-like 1	t=	0.9977	-1.037
	$2^{-\Delta\Delta CT}$ estimates	0.696	1.458
	p(t)	0.3476	0.33
Cyp6-like 2	t=	0.1103	-1.436
	$2^{-\Delta\Delta CT}$ estimates	0.95	1.949
	p(t)	0.9149	0.189
Cyp6-like 3	t=	0.5699	-1.65
	$2^{-\Delta\Delta CT}$ estimates	0.799	1.917
	p(t)	0.5844	0.1375

Cyp6-like 4	t=	1.413	-0.782
	$2^{-\Delta\Delta CT}$ estimates	0.545	1.4
	p(t)	0.1954	0.4565
Cyp6-like 5	t=	2.9869	-1.463
	$2^{-\Delta\Delta CT}$ estimates	0.281	1.861
	p(t)	0.0174	0.1816
Cyp6CM1	t=	0.7423	0.0869
	$2^{-\Delta\Delta CT}$ estimates	0.7306	0.964
	p(t)	0.4791	0.9329
Cyp6-like EE600001	t=	1.2699	-1.237
	$2^{-\Delta\Delta CT}$ estimates	0.625	1.579
	p(t)	0.2398	0.2513
BtGST1	t=	0.4844	-1.749
	$2^{-\Delta\Delta CT}$ estimates	0.847	1.819
	p(t)	0.6411	0.1185
BtGST2	t=	1.6493	-1.932
	$2^{-\Delta\Delta CT}$ estimates	0.531	2.1
	p(t)	0.1377	0.0894
BtGST3	t=	1.2856	-1.262
	$2^{-\Delta\Delta CT}$ estimates	0.559	1.77
	p(t)	0.2346	0.2426
Cyp6-like	t=	1.9353	-1.046
	$2^{-\Delta\Delta CT}$ estimates	0.6001	1.3176
	p(t)	0.089	0.3263

UDP-GT	t=	1.1046	0.2337
	$2^{-\Delta\Delta CT}$ estimates	0.531	0.875
	p(t)	0.3014	0.8211

Quercetin

Gene	Value	MEAM1 (S+P) / (S)	MED (S+P) / (S)
COE1	t=	0.3359	1.0458
	$2^{-\Delta\Delta CT}$ estimates	0.858	0.621
	p(t)	0.7456	0.3262
COE2	t=	0.8803	-0.989
	$2^{-\Delta\Delta CT}$ estimates	0.743	1.397
	p(t)	0.4044	0.3516
Cyp4-like 1	t=	1.3353	-1.051
	$2^{-\Delta\Delta CT}$ estimates	0.625	1.448
	p(t)	0.2185	0.3241
Cyp4-like2	t=	-0.61	-1.358
	$2^{-\Delta\Delta CT}$ estimates	1.276	1.72
	p(t)	0.5586	0.2117
Cyp4-like 4	t=	1.1789	-1.752
	$2^{-\Delta\Delta CT}$ estimates	0.554	2.41
	p(t)	0.2723	0.1179
Cyp4-like 5	t=	0.8108	0.1654
	$2^{-\Delta\Delta CT}$ estimates	0.748	0.943

	p(t)	0.4409	0.8727
Cyp6-like 1	t=	1.4821	-3.232
	$2^{-\Delta\Delta CT}$ estimates	0.57	3.408
	p(t)	0.1766	0.012
Cyp6-like 2	t=	1.4729	0.1379
	$2^{-\Delta\Delta CT}$ estimates	0.542	0.944
	p(t)	0.179	0.8937
Cyp6-like 3	t=	1.2309	1.1629
	$2^{-\Delta\Delta CT}$ estimates	0.493	0.513
	p(t)	0.2533	0.2784
Cyp6-like 4	t=	1.5999	-0.418
	$2^{-\Delta\Delta CT}$ estimates	0.527	1.183
	p(t)	0.1483	0.6866
Cyp6-like 5	t=	1.1324	-2.470
	$2^{-\Delta\Delta CT}$ estimates	0.679	2.333
	p(t)	0.2902	0.0270
Cyp6CM1	t=	0.1582	0.4746
	$2^{-\Delta\Delta CT}$ estimates	0.86	0.636
	p(t)	0.8782	0.6477
Cyp6-like EE600001	t=	1.8342	0.6633
	$2^{-\Delta\Delta CT}$ estimates	0.406	0.7219
	p(t)	0.104	0.5258
BtGST1	t=	0.9911	0.0906
	$2^{-\Delta\Delta CT}$ estimates	0.718	0.97

	p(t)	0.3507	0.93
BtGST2	t=	0.3986	-1.296
	$2^{-\Delta\Delta CT}$ estimates	0.7869	2.1795
	p(t)	0.7006	0.2312
BtGST3	t=	0.3102	0.8391
	$2^{-\Delta\Delta CT}$ estimates	0.888	0.724
	p(t)	0.7644	0.4258
Cyp6-like	t=	0.3133	0.7419
	$2^{-\Delta\Delta CT}$ estimates	0.7661	0.532
	p(t)	0.762	0.4794
UDP-GT	t=	0.2186	0.1257
	$2^{-\Delta\Delta CT}$ estimates	0.839	0.904
	p(t)	0.8324	0.9031

Table S2 Comparisons of constitutive expression levels of detoxification genes between the MED and MEAM1 species for each gene separately**.** Detailed ΔΔCT values and statistics of the ΔCT method. Genes were considered significantly over- or under-transcribed when the ΔCT values of RNA samples from MED were different from ΔCT values of RNA samples from MEAM1 at $P \leq 0.05$.

Gene	Value	MED (S) / MEAM1 (S)
COE1	*F*=	0.678
	$2^{-\Delta\Delta CT}$ estimates	1.19
	p(*F*)	0.415
COE2	*F*=	13.43
	$2^{-\Delta\Delta CT}$ estimates	0.48
	p(*F*)	0.0007
Cyp4-like 1	*F*=	7.93
	$2^{-\Delta\Delta CT}$ estimates	1.85
	p(*F*)	0.0075
Cyp4-like2	*F*=	29.43
	$2^{-\Delta\Delta CT}$ estimates	7.73
	p(*F*)	<0.0001
Cyp4-like 4	*F*=	1.792
	$2^{-\Delta\Delta CT}$ estimates	0.76
	p(*F*)	0.1883
Cyp4-like 5	*F*=	0.2992
	$2^{-\Delta\Delta CT}$ estimates	0.74
	p(*F*)	0.5874

Cyp6-like 1	*F*=	7.76
	$2^{-\Delta\Delta CT}$ estimates	2.16
	p(*F*)	0.0081
Cyp6-like 2	*F*=	5.438
	$2^{-\Delta\Delta CT}$ estimates	1.82
	p(*F*)	<0.0248
Cyp6-like 3	*F*=	2.817
	$2^{-\Delta\Delta CT}$ estimates	2.28
	p(*F*)	0.100
Cyp6-like 4	*F*=	1.051
	$2^{-\Delta\Delta CT}$ estimates	0.77
	p(*F*)	0.3115
Cyp6-like 5	*F*=	0.723
	$2^{-\Delta\Delta CT}$ estimates	1.22
	p(*F*)	0.400
Cyp6CM1	*F*=	5.459
	$2^{-\Delta\Delta CT}$ estimates	1.90
	p(*F*)	0.0246
Cyp6-like EE600001	*F*=	4.633
	$2^{-\Delta\Delta CT}$ estimates	1.69
	p(*F*)	0.0375
BtGST1	*F*=	19.325
	$2^{-\Delta\Delta CT}$ estimates	2.07
	p(*F*)	<0.0001

BtGST2	*F*=	0.0052
	$2^{-\Delta\Delta CT}$ estimates	0.95
	p(*F*)	0.822
BtGST3	*F*=	14.768
	$2^{-\Delta\Delta CT}$ estimates	1.85
	p(*F*)	0.0004
Cyp6-like	*F*=	1.608
	$2^{-\Delta\Delta CT}$ estimates	1.25
	p(*F*)	0.212
UDP-GT	*F*=	174.03
	$2^{-\Delta\Delta CT}$ estimates	0.04
	p(*F*)	<0.0001

Table S3 List of detoxification genes, accession numbers, primers and product sizes used in qRT-PCR analyses.

Gene	Primers 5'→ 3'	Accession number	Product (bp)
BtGST1	CGTTTCATCTTTGCTTATGCCA<br>TCCGTCGATGTCCAAAACGGG	Rauch and Nauen, 2004	120
BtGST2	GGCGGCTATTTAGTGAACG<br>AAACGACCCACAAGTCCG	JF323035	52
BtGST3	GTCTCGTATGCTATCACCGTGT<br>CATCATGACAATTGTTTCTCTTGTT	JF323036	145
COE1	ATGTCAAACCCTGATCAACCA<br>ACTGTAGTAAGTTTGACCCAAG	EF675184	93
COE2	TAGATTCAGATGGACGGCTC<br>CCCTGGCGATCAAATGGGTT	EF675186	110
CYP4-like 1	TCGACCCTGAACGCTTCAC<br>AGCAGCGAAAGGAGCGTAAG	EU344873	71
CYP4-like 2	TGGCCGAACCCTGAAAAGT<br>GCGTAAGGGTGCCTTTTTGA	EU344874	71
CYP4-like 4	CACTAGAAATGAAATACTTGGAAAGGTGTA<br>TTGTAGCTCCCTGGCAATGAT	EU344876	86
CYP4-like 5	TGTAGACAAGTTCCTAAGGACACAAATT<br>AGACATCCAACGTCACGAGAGA	EU344877	76
CYP6CM1	GGCAAGTTCGGGGTCGCGG<br>TCCGGCTTGTCCTCCGAATGG	Q- EU344879<br>B- EU642555	134
CYP6-like 1	CATGCAGTATCTAGAGCAGGTCGTA<br>TGCAGAATCTGAACAAGAATCCA	EU344878	77

CYP6-like 2	AACACGGCGAGGAAAACATC<br>TCTCGTTGAAAACCATTTCCAAT	EU344880	69
CYP6-like 3	GAGCGGATATGTACGAAAGCCT<br>AGGATCTGGAAAGAACTGGGC	EU344881	120
CYP6-like 4	TGGAGGAAACTCTGCGGAAA<br>AATTCGTAAGCCTTCGTGCATAC	EU344882	70
CYP6-like 5	CATGATGGAGACCGTAGACAGAAA<br>TTCCGGATACGAATGTTGT	EU344883	69
CYP6-like EE600001	CTTGAGTCTGATGAGGAAGT<br>ACCACATCGAATCGCTGAA	EE600001	107
CYP6-like	CAATGCCCAGCAACTCTACG<br>CACCTTGTCTTGGAGGTAG	JN165263	74
UDP-GT	AGCACCCAGGAGTCCATCTA<br>GCAATTCCTGCCTTGACCATT	Leshkowitz et al., 2005<br>BT-TYLCV-017-1-E1-T3_E01	100
Actin	TGATGATACCGTGCTCGATGG<br>TCAGGGTGTAATGGTCGGTA	KC16211	110

Leshkowitz, D. *et al*. Whitefly (*Bemisia tabaci*) genome project: analysis of sequenced clones from egg, instar, and adult (viruliferous and non-viruliferous) cDNA libraries. *BMC Genomics* **7**, 79 (2006).

Rauch, N. & Nauen, R. 2004. Characterization and molecular cloning of a glutathione S-transferase from the whitefly *Bemisia tabaci* (Hemiptera: Aleyrodidae). *Insect biochem. Mol. Boil.* **34**, 321-329 (2004).

Table S4 Minimum Information for Publication of Quantitative Real-Time PCR Experiments (MIQE) data.

ITEM TO CHECK	IMPORTANCE	CHECKLIST
**EXPERIMENTAL DESIGN**		
Definition of experimental and control groups	**E**	**Experimental group:** *Bemisia tabaci* MEAM1 and MED species, fed on sucrose artificial diet with phytotoxins (caffeine,nicotine,quercetin,flavone,indole 3 carbinol, allyl isothiocyanate). **Control group:** *Bemisia tabaci* MEAM1 and MED species, fed on sucrose artificial diet without phytotoxins.
Number within each group	**E**	n=at least 3 for each treatment and control
Assay carried out by core lab or investigator's lab?	D	
Acknowledgement of authors' contributions	D	
**SAMPLE**		
Description	**E**	*Bemisia tabaci* (whole organism)
Volume/mass of sample processed	D	
Microdissection or macrodissection	**E**	Microdissection of mid-gut was performed as described in Materials and Methods.
Processing procedure	**E**	50 individuals where collected in 1.5ml tubes and homogenized with TRIzol reagent (Invitrogen).
If frozen - how and how quickly?	**E**	Samples were stored at -70°C after homogenization if not processed immidiately.
If fixed - with what, how quickly?	**E**	Not fixed.

Sample storage conditions and duration (especially for FFPE samples)	**E**	Frozen homogenized samples where stored at -70°C.
**NUCLEIC ACID EXTRACTION**		
Procedure and/or instrumentation	**E**	Total RNA was extracted using the TRIzol Reagent (Invitrogen) following manufacture's protocol. Homogenization of samples was performed in 1.5 ml tubes using 0.2 ml of TRIzol Reagent per 50 individuals. Following homogenization, 1/6 of (final volume) chloroform was added and the tubes where centrifuged at 12,000 g for 15 minutes at 4°C. Then the soluble material from the homogenate was moved to an new tube and incubated with 70% volume of 2-propanol for 2 hours at 4°C. After another centrifugation (12,000g for 10 min at 4°C) following ethanol purification, the purified RNA was dissolved in 20 µl DEPC-Treated Water and stored at -70°C.
Name of kit and details of any modifications	**E**	TRIzol Reagent (Invitrogen), following manufacture's protocol.
Source of additional reagents used	D	Chloroform (Bio Lab); 2-propanol (J.T. Baker); Ethanol (Gadot); DEPC (Sigma).
Details of DNase or RNAse treatment	**E**	2µg of RNA was treated with 2µl DNaseI (Promega), in a 15 µl final volume reaction. Digestion of DNA was achieved with 30 minutes incubation at 37°C. The reaction was stopped with 1µl of DNase Stop Solution (Promega) following 10 min incubation at 65°C for inactivation.
Contamination assessment (DNA or RNA)	**E**	-RT (reverse transcription control (without enzyme) were performed in order to assess the absence of DNA in the RNA samples.
Nucleic acid quantification	**E**	RNA concentration was determined by measuring the absorbance at 260 nm.
Instrument and method	**E**	Nanodrop (Thermo scientific).
Purity (A260/A280)	D	RNA purity was determined by measuring the absorbance ratio 260/280.
Yield	D	
RNA integrity method/instrument	**E**	The RNA integrity was assessed by the 18S and 28S band intensity ratio after 1% agarose gel electrophoresis, visualized by ethidium bromide staining.

RIN/RQI or Cq of 3' and 5' transcripts	**E**	Not applicable.
Electrophoresis traces	D	
Inhibition testing (Cq dilutions, spike or other)	**E**	The standard curve has been considered sufficient to rule out the presence of inhibitors of reverse-transcription activity or PCR, also taking into account the high quality of starting RNAs.
**REVERSE TRANSCRIPTION**		
Complete reaction conditions	**E**	First strand cDNA was synthesized by Superscript II (Invitrogen) using 1 µg total RNA and an Oligo-dT primer according to the manufacturer's instructions. The -RT samples did not contain the enzyme.
Amount of RNA and reaction volume	**E**	1µg of RNA was used in total reaction of 20 µl.
Priming oligonucleotide (if using GSP) and concentration	**E**	Oligo dT - 20pmol/µl
Reverse transcriptase and concentration	**E**	Superscript II (200U/µl) (Invitrogen) - 1 µl.
Temperature and time	**E**	50 min at 42ºC followed by 70ºC for 15 min inactivation.
Manufacturer of reagents and catalogue numbers	D	
Cqs with and without RT	D*	
Storage conditions of cDNA	D	-70°C. Diluted cDNA (1:20) in -20°C.
**qPCR TARGET INFORMATION**		
If multiplex, efficiency and LOD of each assay.	**E**	Not applicable.
Sequence accession number	**E**	See supplementary table S3.
Location of amplicon	D	
Amplicon length	**E**	See supplementary table S3.

In silico specificity screen (BLAST, etc)	**E**	Not applicable.
Pseudogenes, retropseudogenes or other homologs?	D	
Sequence alignment	D	
Secondary structure analysis of amplicon	D	
Location of each primer by exon or intron (if applicable)	**E**	Not applicable.
What splice variants are targeted?	**E**	No genomic information avilable.
**qPCR OLIGONUCLEOTIDES**		
Primer sequences	**E**	See supplementary table S3.
RTPrimerDB Identification Number	D	
Probe sequences	D**	
Location and identity of any modifications	**E**	No modification where made.
Manufacturer of oligonucleotides	D	Sigma.
Purification method	D	Desalted.
**qPCR PROTOCOL**		
Complete reaction conditions	**E**	PCR reactions were performed in a GeneAmp 7300 (Applied Biosystems) using SYBR® Green Mix (ABgene) in final volume of 18 µl. The reaction mix containing: 9 µl Absolute QPCR SYBR Green Mix (ABgene), 150 nM or 250 nM forward and reverse primers and 2 µl of cDNA. PCR thermal conditions consisted one cycle of 50 °C for 2 min, one cycle of 95 °C for 2 min, followed by 40 cycles of 95 °C for 15 sec and 60 °C for 1 min.

Reaction volume and amount of cDNA/DNA	**E**	Reaction volume: 18 µl; amount of cDNA: 2 ul of 1/20 dilution.
Primer, (probe), Mg++ and dNTP concentrations	**E**	Primer concentration: **Actin** - 250nM; **RPLA** - 250nM; **COE1** - 250nM; **COE2** - 150nM; **Cyp4like1** - 150nM; **Cyp4like2** - 150nM; **Cyp4like4** - 250nM; **Cyp4like5** - 250nM; **Cyp6like1** - 150nM; **Cyp6like2** - 150nM; **Cyp6like3** - 250nM; **Cyp6like4** - 250nM; **Cyp6like5** - 150nM; **Cyp6CM1** - 250nM; **Cyp6likeEE600001** - 150nM; **BtGST1** - 250nM; **BtGST2** - 250nM; **BtGST3** - 250nM; **Cyp6-like** - 250nM; **UDP-GT** - 250nM; 100 µL MgCl2 (1M) and dNTPs are included in the mix.
Polymerase identity and concentration	**E**	Thermo-Start DNA Polymerase; concentration is not specified.
Buffer/kit identity and manufacturer	**E**	ABgene (Life thechnologies).
Exact chemical constitution of the buffer	D	
Additives (SYBR Green I, DMSO, etc.)	**E**	SYBER Green as part of the mix.
Manufacturer of plates/tubes and catalog number	D	
Complete thermocycling parameters	**E**	PCR thermal conditions consisted one cycle of 50ºC for 2 min, one cycle of 95ºC for 2 min, followed by 40 cycles of 95ºC for 15 sec and 60ºC for 1 min.
Reaction setup (manual/robotic)	D	
Manufacturer of qPCR instrument	**E**	GeneAmp 7300 (Applied Biosystems).
**qPCR VALIDATION**		
Evidence of optimisation (from gradients)	D	
Specificity (gel, sequence, melt, or digest)	**E**	Melting curves were conducted to all samples to check for specific gene amplification. Moreover, no template controls (NTC - no cDNA in PCR) were run for each gene to detect unspecific amplification and primer dimerization.

For SYBR Green I, Cq of the NTC	**E**	When present, the signal amplification plot was very late (Cq>34) therefore there was a high diffrence between the negative control and all the cDNA samples.
Standard curves with slope and y-intercept	**E**	**Cyp6like**: slope(-3.34) y intercept (20.3); **Cyp6like5**: slope(-3.4) y intercept (21.24); **UDP-GT**: slope(-3.53) y intercept (17.47); **Cyp6like2**: slope(-3.37) y intercept (19.33); **COE2**: slope(-3.43) y intercept (16.48); **Cyp6like3**: slope(-3.37) y intercept (17.99); Bt**GST1**: slope(-3.57) y intercept (21.26); **BtGST2**: slope(-3.38) y intercept (30.02); **BtGST3**: slope(-3.46) y intercept (20.19); **Cyp4like1**: slope(-3.47) y intercept (21.51); **Cyp4like2**: slope(-3.49) y intercept (17.85); **Cyp4like4**: slope(-3.4) y intercept (17.19); **Cyp4like5**: slope(-3.4) y intercept (23.83); **Cyp6like1**: slope(-3.4) y intercept (17.36); **Cyp6like4**: slope(-3.39) y intercept (20.04); **Cyp6CM1**: slope(-3.51) y intercept (19.52); **Cyp6EE600001:** slope(-3.67) y intercept (23.4); **COE1**: slope(-3.53) y intercept (23.46); **Actin:** slope(--3.32) y intercept (14.78); **RPLA:** slope(-3.56) y intercept (16.52).
PCR efficiency calculated from slope	**E**	**Cyp6like** (0.99) **Cyp6like5** (0.97) **UDP-GT** (0.92) **Cyp6like2** (0.98) **COE2** (0.96) **Cyp6like3** (0.98) **BtGST1** (0.91) **BtGST2** (0.98) **BtGST3** (0.95) **Cyp4like1** (0.94) **Cyp4like2** (0.93) **Cyp4like4** (0.97) **Cyp4like5** (0.97) **Cyp6like1** (0.97) **Cyp6like4** (0.97) **Cyp6CM1** (0.93) **Cyp6EE600001** (0.87) **COE1** (0.92) **Actin** (1) **RPLA** (0.91).
Confidence interval for PCR efficiency or standard error	D	
r2 of standard curve	**E**	**Cyp6like** (0.98), **Cyp6like5** (0.99), **UDP-GT** (0.96), **Cyp6like2** (0.99), **COE2** (1), **Cyp6like3** (0.99), **BtGST1** (0.99), **BtGST2** (0.96), **BtGST3** (1), **Cyp4like1** (0.98), **Cyp4like2** (0.96), **Cyp4like4** (0.99), **Cyp4like5** (0.98), **Cyp6like1** (0.99), **Cyp6like4** (0.98), **Cyp6CM1** (0.99), **Cyp6EE600001** (0.98), **COE1** (0.97), **Actin** (1), **RPLA** (0.98),
Linear dynamic range	**E**	The linear dynamic range was determined by a standard curve of the cDNA in the following dilutions 1:10, 1:20, 1:40, 1:80, 1:160, 1:320
Cq variation at lower limit	**E**	The assay did not reach the limit of detection.
Confidence intervals throughout range	D	

Evidence for limit of detection	**E**	The assay did not reach the limit of detection.
If multiplex, efficiency and LOD of each assay.	**E**	Not applicable.
**DATA ANALYSIS**		
qPCR analysis program (source, version)	**E**	Applied Biosystems 7300 Real Time PCR System V1.4.
Cq method determination	**E**	The threshold is determined using the Amplification-based Threshold method. The threshold is used to specify Cq values of samples.
Outlier identification and disposition	**E**	In general, no biological replicates where omitted only in cases of outlier box plot analysis.
Results of NTCs	**E**	When present the signal amplification plot was very late (Cq>34) therefore there was a high diffrence between the negative control and all the cDNA samples.
Justification of number and choice of reference genes	**E**	We used a single gene, actin (accession number KC16211) as our reference gene. We analyzed the stability of actin and five other genes in our reported experimental system (Cyp4-like 2, Cyp6-like 3, Cyp6CM1, UDP-GT and BtGST3) which did not respond to any of the reported phytotoxins treatments (Figure 1a), using the programs geNorm and Normfinder. In Normfinder the stability value was 0.33 and was ranked first together with Cyp6-like 3 (same stability value). BtGST3 was ranked third with stability value of 0.52. The Normfinder stability value of 0.33 is comparable to that of recommended reference genes in other B. tabaci papers. Moreover, using the pair Cyp6-like 3 and actin only reduced the stability value to 0.27, suggesting that adding an additional reference gene will not significantly improve the analysis. In the geNorm analysis, the M stability values of actin (step 1) ranged between 0.012-0.0720 which is below 0.15 and therefore indicates that an additional reference gene will not significantly improve normalization.
Relevant literature of reference genes	**D**	Collins, C., Patel, V. B., Colvin, J., Bailey, D. & Seal, S. Identification and evaluation of suitable reference genes for gene expression studies in the whitefly *Bemisia tabaci* (Asia I) by reverse transcription quantitative real-time PCR. *J. Insect Sci.* **14**, 63 (2014).

		Li, R. *et al*. Reference gene selection for qRT-PCR analysis in the sweetpotato whitefly, *Bemisia tabaci* (Hemiptera: Aleyrodidae). *PLoS One* **8**, e53006 (2013).<br>Liang, P., Guo, Y., Zhou, X. & Gao, X. Expression profiling in *Bemisia tabaci* under insecticide treatment: indicating the necessity for custom reference gene selection. *PLoS One* **9**, e87514 (2014).<br>Su, Y. L. *et al*. (2013). Selection of endogenous reference genes for gene expression analysis in the Mediterranean species of the *Bemisia tabaci* (Hemiptera: Aleyrodidae) complex. *J. Econ. Entomol.* **106**, 1446-1455 (2013).
Description of normalisation method	**E**	Normalization was done using reference gene (ΔCT method).
Number and concordance of biological replicates	D	3 biological replicates where done.
Number and stage (RT or qPCR) of technical replicates	**E**	3 technical replicates per biological replicate.
Repeatability (intra-assay variation)	E	Not applicable.
Reproducibility (inter-assay variation, %CV)	D	
Power analysis	D	
Statistical methods for result significance	**E**	Gene induction data were analyzed by one-way ANOVA model (diet-type, 'sucrose only' versus sucrose plus phytotoxin, as the main effect) for each species, gene and phytotoxin separately.
Software (source, version)	E	JMP statistical software version 10 (SAS Institute, USA).
Cq or raw data submission using RDML	**D**	
